# Phytochemical drivers of insect herbivory: a functional toolbox to support agroecological diversification

**DOI:** 10.1098/rsos.240890

**Published:** 2024-07-17

**Authors:** Jeremy Grosjean, Foteini G. Pashalidou, Aude Fauvet, Aurore Baillet, Alan Kergunteuil

**Affiliations:** ^1^ Université de Lorraine, LAE, INRAE, 54000 Nancy, France; ^2^ Platform of Structural and Metabolomics Analyses, SF4242, EFABA, Lorraine University, Vandoeuvre-les-Nancy, France; ^3^ Agronomie, INRAE, AgroParisTech, Université Paris-Saclay, Palaiseau, France; ^4^ Terres Inovia, 9 rue de la Vologne, 54520 Laxou, France; ^5^ INRAE, PSH, 84000 Avignon, France

**Keywords:** associational resistance, bottom-up, *Brassica napus*, mixed crop, plant defence, plant metabolism

## Abstract

Plant metabolism is a key feature of biodiversity that remains underexploited in functional frameworks used in agroecology. Here, we study how phytochemical diversity considered at three organizational levels can promote pest control. In a factorial field experiment, we manipulated plant diversity in three monocultures and three mixed crops of oilseed rape to explore how intra- and interspecific phytochemical diversity affects pest infestation. We combined recent progress in metabolomics with classic metrics used in ecology to test a box of hypotheses grounded in plant defence theory. According to the hypothesis of ‘phytochemically mediated coevolution’, our study stresses the relationships between herbivore infestation and particular classes of specialized metabolites like glucosinolates. Among 178 significant relationships between metabolites and herbivory rates, only 20% were negative. At the plant level, phytochemical abundance and richness had poor predictive power on pest regulation. This challenges the hypothesis of ‘synergistic effects*’*. At the crop cover level, in line with the hypothesis of ‘associational resistance’, the phytochemical dissimilarity between neighbouring plants limited pest infestation. We discuss the intricate links between associational resistance and bottom-up pest control. Bridging different levels of organization in agroecosystems helps to dissect the multi-scale relationships between phytochemistry and insect herbivory.

## 1. Background

Over the last few decades, trait-based analyses have helped to revisit empirical approaches in agroecological diversification [1]. Plant morphological traits, such as height, biomass, root length or floral depth, have prompted functional frameworks used to reduce plant competition [2,3], improve nutrient cycling [4,5] or increase the presence of biological control agents [6,7]. In this context, phytochemistry offers a vast repository of functional traits that open new avenues for developing complementary frameworks that improve pest control. Recently, Collaza *et al*. [8] have stressed the importance of phytochemical diversity produced by floral resources to promote conservation biological control in agroecosystems. Similarly, major developments in chemical ecology provide interesting elements to promote bottom-up pest control. On the one hand, there is a growing interest in aggregating phytochemical traits underpinning antiherbivore plant defences in functional ecology based on breakthroughs in metabolomic and bioinformatics [9]. On the other hand, ecologists strive to relate different organization levels where phytochemistry can be considered to address appropriately different hypotheses of plant defence theory [10]. Because this research line helps to dissect a suite of mechanisms involved in bottom-up pest control, it provides a sound approach to harness the potential of phytochemistry in agroecological research.

The role of plant metabolism in pest control has long revolved around the antiherbivore properties of particular compounds. Primary plant metabolites such as hormones [11], amino acids [12] or lipids [13] are intricately related to herbivore attacks. In addition, specialized metabolites with toxic properties against herbivores have been of first importance in the emergence of the idea of plant–insect coevolution [14,15]. The so-called ‘phytochemically mediated coevolutionary’ hypothesis has sometimes led to the warlike analogy of plant–insect arms race based on the continuous evolution of plant chemical defences and insect detoxifying processes [16–18]. Across the plant kingdom, some classes of specialized metabolites are widely recognized for their influence on pest regulation (see [19], for furocoumarins; [20], for cardenolides; and [21], for iridoid glycosides). In wild brassicaceous plants, the diversity of glucosinolates offers a rich source of metabolites with antiherbivore properties against generalist pests [22]. However, specialist herbivores can use defensive compounds like glucosinolates as feeding stimulants [23] or for their own defences [24,25]. These versatile effects of glucosinolates on generalist and specialist pests feed an active debate about the interest in conserving them in modern cultivars [26,27].

Beyond the antiherbivore properties of particular compounds, a turning point in chemical ecology was reached with functional analyses of phytochemical traits on two higher organizational levels. First, on the plant level, the maintenance of coexpression of multiple defensive compounds remains an open question [28]. In this context, Richards *et al*. [29] have reviewed the hypothesis of ‘synergistic effects’, suggesting that several metabolites in a mixture affect concomitantly different functions of insects. Despite challenges in data computation, the numerous examples supporting synergistic effects encourage us to study how pests are affected by the entire set of defensive compounds produced by individual plants [30]. Second, on the community level, chemical ecologists attempt to scale up individually centred mechanisms towards ecological outcomes like dynamics of insect populations [31–33]. This perspective is promising to test the ‘associational resistance’ hypothesis coined by Tahvanainen & Root [34], who assumed that phytochemical dissimilarity between neighbouring plants reduces the colonization of crop covers by pests. High-throughput metabolomics has opened a new era for plant ecologists interested in the mechanisms underlying associational resistance in plant communities like grasslands [35,36] or tropical forests [37,38]. Transposing this question into agroecosystems could help develop innovative strategies for the diversification of agroecological crop cover.

To do this, we monitored herbivore infestation in oilseed rape (*Brassica napus*) attacked by the cabbage stem flea beetle, *Psylliodes chrysocephala* (Coleoptera:Chrysomelidae), a specialist herbivore that has recently evolved resistances against pyrethroids [39]. As a result, this pest became a major threat to brassicaceous crops in several European countries [40]. Here, we estimate pest infestation in six crop covers where we manipulate plant diversity in three commercialized cultivars grown alone and three mixed crop covers. Based on agroecological consistency, mixed crops were designed with a particular type of companion plants, i.e. trap plants [41], to explore intra- and interspecific crop diversification. To test whether and how plant diversity affects phytochemical diversity, we brought together cutting-edge technologies in mass spectrometry [42], recent developments in open-source and reproducible analyses of metabolomic data [43,44] and community-wide initiatives that enable molecular network analyses from public libraries of mass spectra [45]. In addition, we used recent ideas to study phytochemical diversity at the plant and community levels based on classic metrics in ecology [10].

In the present study, we aimed to (i) identify natural compounds driving pest infestation in modern cultivars of brassicaceous crops, (ii) test if individual phytochemical makeup reduces pest infestation on focal plants, and (iii) test if phytochemical abundance and diversity affect pest infestation at the crop scale. We embedded our work within plant defence theory to test the hypotheses of ‘phytochemically mediated coevolution’, ‘synergistic effect’ and ‘associational resistance’ at the compound, plant and community scales, respectively. The integration of these three hypotheses predicting herbivory at different organization levels may help to address important questions about the heterogeneity of herbivory observed in nature. From an applied perspective, we hope that our study will extend functional frameworks used to reason biodiversity-based management in cropping systems less reliant on insecticides.

## 2. Material and methods

### 2.1. Study system and experimental design

**Figure 1.**
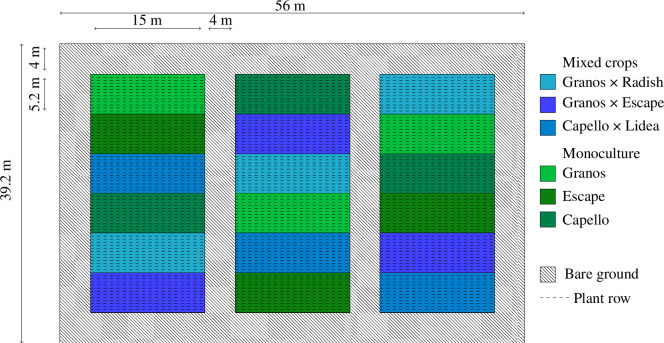
Field experimental setup. The field was surrounded by 5 ha of oilseed rape. In mixed crops, the proportions of trap plants are 14%, 7% and 7% of radish, ‘Escape’ and ‘Lidea’, respectively.

In a suite of experiments coordinated by the technical institute Terres Inovia, we co-designed a factorial experiment with six different crop covers. Each of the six crop covers was sown on a 5.2 × 15 m^2^ area and replicated in three spatial blocks separated by bare ground (4 m) according to a random block design (figure 1). The experimental setup was surrounded by a 5 ha field of oilseed rape to limit bordure effects in pest colonization patterns. Three monocultures of oilseed rape (*B. napus*) were used to compare phytochemical diversity and pest infestation in two target cultivars (‘Granos’ and ‘Capello’) and one cultivar commercialized as a trap plant (‘Escape’). In three additional mixed covers, we modulated intra- and interspecific diversity. First, to manipulate intraspecific diversity, we associated target cultivars (‘Granos’ and ‘Capello’) with two oilseed rape cultivars commercialized as trap plants (‘Escape’ and ‘Lidea’). These two cultivars are known to support a greater number of *P. chrysocephala* larvae even if the underlying mechanisms remain an open question. In this study, Terres Inovia developed the two tested combinations of target and trap cultivars, that is ‘Granos × Escape’ and ‘Capello × Lidea’, as a new strategy based on their agronomic potential. Second, for the manipulation of interspecific crop covers, the oilseed rape cultivar ‘Granos’ was mixed with radish ‘Daikon’ (*Raphanus sativus* var. *longipinnatus*) according to the classic practices used in the diversification of heterospecific oilseed rape crop cover. Radish is considered trap plant for several oilseed rape pests, such as shown for flea beetles [46]. Overall, in the three mixed crops, we used reasonable proportions of trap plants from agronomic perspectives (15% for radish, 7% for ‘Escape’ and ‘Lidea’ cultivars). Based on plant morphological differences within ‘Granos × Radish’ and ‘Granos × Escape’ mixed crops, we were able to distinguish radish and ‘Escape’ from ‘Granos’. However, owing to unforeseen phenotype similarity between ‘Lidea’ and ‘Capello’, we were unable to adequately discriminate both cultivars in the mixed crop ‘Capello × Lidea’. Hence, this crop cover was only used to address the effects of the phytochemical diversity considered on the crop scale.

### 2.2. Monitoring pest infestation in different cultivars

After adults of *P. chrysocephala* colonize crops during autumn, their flight muscles atrophy and individuals mate. Females lay eggs in soil close to root collards. Following hatching, larvae move to plants where they mine stems, causing severe damage all along overwintering [47]. On 3 December 2021, after all *P. chrysocephala* eggs hatched and the larvae penetrated the stems, we sampled 147 plants belonging to the different cultivars described above. In the field, the biomass of the shoots was measured immediately and stored at 4°C before being brought to the laboratory. The shoots were transferred to Berlese traps in a room kept at a constant temperature (18°C). In brief, plants were placed on a metal grid (1 cm² mesh size) on a container filled with liquid (water with 1 ml l^−1^ of odourless soap used as surfactant) [48]. During two weeks, we counted twice a week the number of larvae that fell in the liquid. On 17 December, we summed the total number of larvae of *P. chrysocephala* that were collected per plant.

### 2.3. Chemical analyses in different cultivars

#### 2.3.1. Untargeted metabolomic extraction

For each of the 147 plants sampled, two leaves with the same phenological stage were used to extract metabolites according to an untargeted approach to capture a fraction of plant metabolism as vast as possible. We developed a cold-temperature solid–liquid extraction by maintaining samples below 4°C throughout the extraction process to conserve glucosinolates. In general, cold temperature limits spurious changes in metabolites owing to biochemical reactions or thermal lability that could affect the integrity of metabolomic profiles [49,50]. In the specific case of glucosinolates, some studies confirmed that inorganic solvents maintained at cold temperatures prevent their degradation by the myrosinase enzyme [51–53]. In brief, shoots were crushed in mortar filled with liquid nitrogen and 215.35 ± 10.38 mg of fresh mass was transferred to a 2 ml Eppendorf tube. Then, a double extraction was performed after we peaked the tubes with an internal standard (taxifolin). For the first extraction, we used 1 ml of methanol : water (70 : 30) to retrieve apolar fractions of the metabolome. The samples were sonicated for 10 min using a sweep mode to ensure variations in frequencies to break the cell wall and favour the release of metabolites. Tubes were agitated for 24 h and centrifuged (10 min at 13 200 rpm). The supernatant was collected (750 µl) and pellets were used for the second extraction in 1 ml of methanol : water (30 : 70) to retrieve polar metabolites. The tubes were agitated for 5 h and centrifuged (10 min at 13 200 rpm). The second supernatant (750 µl) was merged with the first one. For each sample, 100 µl was filtered (RC membrane, 0.2 µm) and transferred to a UHPLC vial. The samples were stored at −20°C before chemical analyses.

#### 2.3.2. Chromatographic separation and tandem mass spectrometry

Liquid chromatographic analyses were performed on a Vanquish UHPLC system equipped with a quaternary pump, an autosampler and a temperature-controlled column oven. We used an XB-C18 Kinetex column (150 × 2.1 mm, 2.6 μm) (Phenomenex Inc., Torrance, CA, USA) to separate the metabolites contained in the extracts, using a mobile phase gradient composed of water + 0.1% formic acid (A) and methanol + 0.1% formic acid (B) at a flow rate of 200 μl min^−1^, while maintaining the column temperature at 40°C. The elution programme consisted of starting with 1% B for 2 min, then linearly increasing from 1 to 20% B in 8 min, then to 95% B in 10 min. The column was rinsed for 5 min with 95% B and re-equilibrated to the initial conditions for 4 min prior to the next run. The samples were analysed randomly. High-resolution mass spectrometry detection was performed on an Orbitrap IDX mass spectrometer (ThermoFisher Scientific, Bremen, Germany) in positive or negative electrospray ionization (ESI) modes. The capillary voltages were set at 3.5 and 2.5 kV for positive and negative modes. The source gases were set (in arbitrary unit min^−1^) at 40, 8 and 1 (for, respectively, sheath, auxiliary and sweep gas) and the vaporizer temperature was 320°C. Full-scan mass spectra were acquired from 120 to 1200 *m*/*z* at a resolution of 60 000. MS/MS analysis was performed for each sample using the data-dependent acquisition mode for every mass spectrum, a MS/MS spectrum was acquired at a resolution of 15 000 after a higher-energy C-trap dissociation and a collision-induced dissociation of the sixth most intense MS1 signals.

#### 2.3.3. Metabolomic dereplication and standardization

LC–MS data were dereplicated with an ecosystem of open-source bioinformatic tools. Chromatograms were converted to mzML format with MSConvert (v. 3.0.22015) and subsequently processed with the Bioconductor environment in R. Chromatogram peaks were detected with the ‘CentWave’ algorithm developed in the package ‘xcms’ [54]. After optimization, we used the following parameters: ppm = 5; peak width = [5, 40 s]; noise = 1 × 10^5^ and snthresh = 10. The peaks were aligned with the ‘Obiwarp’ method [55] and grouped according to peak densities [56] (minfrac = 1/3) before performing gap-filling based on the mass-to-charge ratio and retention times (‘FillChromPeaksParam’ function). The resulting metabolomic features were collapsed into pseudospectra of metabolites according to the compounding approach developed by Rainer [57]. From a biological perspective, we argue that this step limits pitfalls in the interpretation of the metabolome since it favours conclusions about metabolites, rather than ionic features deriving from breakdowns formed in ionization sources. For this purpose, we gathered metabolomic features into pseudospectra according to similar retention time (SimilarRtimeParam = 10 s), abundance correlations across samples (threshold = 0.8, transform = log2) and peak shape similarity of EICs within samples (threshold = 0.8, *n* = 2). For each pseudospectrum, we subsequently retrieved the base peak (feature with the highest intensity) for further analysis. In LC–MS with ESI mode, these base peaks are expected to be molecular ions, whether [M − H]^+^, [M − H]^−^ or [M + adduct]^+/−^. After we subtracted 76 contaminants deriving from the analytical system based on blank chromatograms, we teased apart 30 duplicated metabolites detected in both negative and positive modes. Metabolites were considered duplicates when they shared absolute *m*/*z* slices <2 and absolute retention time window <20 s. In addition, we removed 139 metabolites for which no MS2 spectra were acquired for metabolomic annotation. As a result, our metabolomic dataset includes 1074 different metabolites. For each metabolite, we standardized peak areas with fresh biomass used for metabolomic extraction and the area of internal standard (taxifolin) detected in the same sample.

#### 2.3.4. Metabolomic annotation

**Figure 2.**
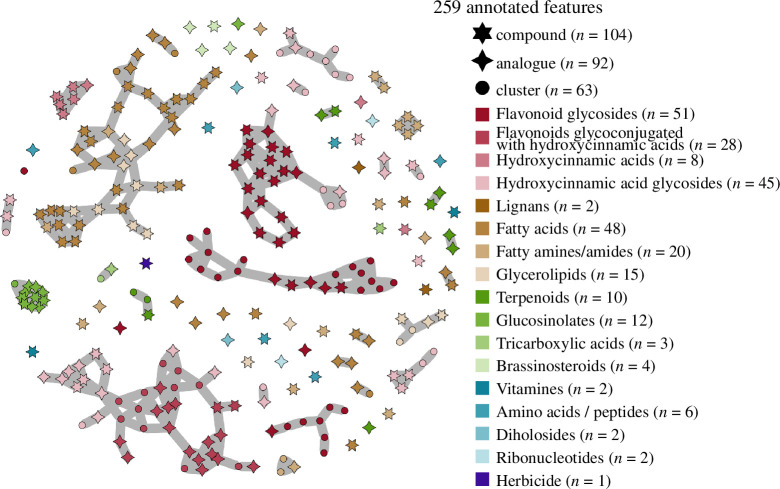
Phytochemical diversity detected in oilseed rape. Metabolomic network depicting the 259 features with chemical assignation among the 1074 features detected. Grey segments between features indicate structural similarities (cosine score >0.7). Comparisons of MS/MS fragments with GNPS libraries enabled us to assign 104 features to specific compounds (six-branched stars) and 155 features to chemical classes when they were analogous to specific compounds (four-branched stars) or when they belong to metabolomic clusters (circles).

We tentatively annotated the 1074 metabolites based on MS2 spectra available on the open community platform Global Natural Products Social Molecular Networking. A molecular network was created with the Feature-Based Molecular Networking (FBMN) workflow [45]. Our metabolomic dataset was filtered by removing all MS/MS fragment ions within ±17 Da of the precursor *m*/*z*. MS/MS spectra were filtered to select the top six fragment ions in the ±50 Da window throughout the spectrum. The precursor ion mass tolerance as well as the MS/MS fragment ion tolerance were set to 0.02 Da. A molecular network was then created with edges displaying a cosine score superior to 0.7 and including more than six matched peaks. In figure 2, this metabolite network was pruned based on the 259 metabolites that were annotated according to three methods. First, 104 metabolites were assigned to specific compounds (cosine score >0.7 and six matched peaks at minimum). Second, 92 metabolites were assigned to chemical classes if they were analogues of known compounds with similar chemical structures (cosine score >0.7 and six matched peaks at minimum) but with different masses (precursor ion values with a maximum difference of 100 Da). Third, 63 metabolites were assigned to chemical classes when they clustered in the FBMN with a group of metabolites belonging to the same chemical class (cosine scores >0.7 and more than six matched peaks). To confirm some annotations, we injected 21 external standards purchased from Sigma Aldrich (Darmstadt, Germany), i.e. 10 glucosinolates (gluconasturtiin, glucoiberin, glucobarbarin, glucoalyssin, gluconapin, glucoraphenin, progoitrin, glucobrassicanapin, glucobrassicin and glucoberteroin) and 11 additional compounds (sophoraflavonoloside, pantothenate, oxidized l-glutathione, chicoric acid, 1-monolinolein, methyl-l-tryptophan, citric acid, ferulic acid, sinapic acid, chlorogenic acid and *p*-coumaric acid).

### 2.4. Data analysis

All analyses were conducted with R v. 4.3.2 [58] using the packages vegan [59], scales [60], randomForest [61], dendextend [62], gplots [63], lme4 [64], merTools [65], car [66] and emmeans [67]. Prior to analyses, the metabolomic dataset was log(*x* + 1) transformed and rescaled to meet the assumption of normality. In metabolomics, rescaling can be considered as an interesting ranking approach since feature abundances are related to chemical structures [9]. For pest infestation, because plants with high biomass generally have more larvae, we standardized the number of larvae recorded in Berlese traps by the fresh biomass of shoots to compute herbivory rate.

#### 2.4.1. Variations of the plant metabolome across cultivars and species

To test the singularity of the plant metabolome between cultivars and species, we performed 1000 random forest analyses (function ‘randomForest’) after we estimated the best number of variables randomly sampled as candidates at each split with the function ‘tunreRF’ (mtry = 128). The features with the highest importance variable measure in 99% of random forest analyses were recovered to perform a hierarchical clustering of plants (function ‘hclust’) based on Euclidean distance matrices and the agglomerative algorithm ‘Ward.D2’. We used a heat map with a pair of dendrograms (function ‘heatmap.2’) to visualize the arrangement of plant and metabolomic clusters.

#### 2.4.2. The effect of natural compounds on herbivory rate

**Figure 3.**
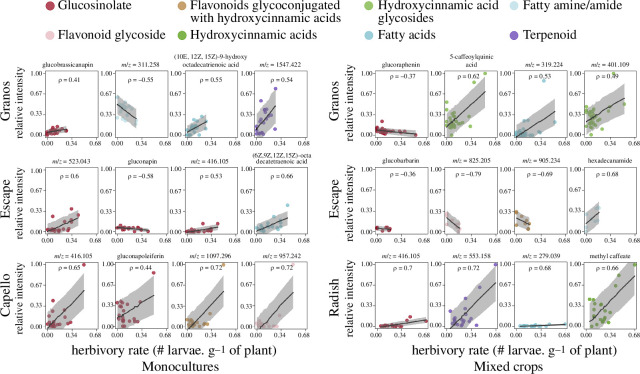
Relationships between pest infestation and natural compounds. Black lines indicate significant mixed linear models (‘block’ as random factor; *p* ≤ 0.05). Grey areas depict prediction intervals (80%) based on simulated models (*n* = 1000). Correlations are given as Pearson coefficients.

For each of the six crop covers, the relationships between herbivory rate and particular metabolites within individual plants were estimated with linear mixed-effect models (function ‘lmer’; spatial block as random effect). For visual purposes, the 178 significant relationships (electronic supplementary material, figure S1) were restricted to 24 to plot figure 3. To do this, we combined two targeted approaches based on glucosinolate and the highest Pearson’s coefficient correlations (function ‘cor.test’) for compounds belonging to other chemical classes. For each relationship, we capture the uncertainty of linear mixed-effect models with prediction interval for the fitted values (function ‘predictInterval’, level = 0.8).

#### 2.4.3. The effect of individual phytochemical makeup on herbivory rate

In addition to the abundance of phytochemical makeup (i.e. sum of relative intensities of compounds), we tested the effect of the richness of phytochemical makeup (i.e. number of compounds)—as recommended by Walker *et al*. [9]—on herbivory rates. For this purpose, we performed linear mixed-effect models (function ‘lmer’; spatial block as random effect) and computed prediction intervals for fitted values (function ‘predictInterval’, level = 0.8).

#### 2.4.4. The effect of crop cover phytochemistry on pest occurrence

Similarly to most studies addressing biodiversity–ecosystem functioning by investigating ecosystem responses based on randomly assembled plant communities [68], we analysed the relationships between pest occurrence and crop cover phytochemistry based on bootstrapped-communities of plants collected in each crop cover. To compute the abundance of phytochemistry per square metre, we summed the relative intensities of compounds detected in plant communities occupying such surface (i.e. 40 plants according to the average square metre density of oilseed rape sown in the six experimental crop covers). Likewise, the phytochemical dissimilarity was estimated with the average abundance-based Jaccard index. While this index is classically used as a proxy of β-diversity in ecology, more particularly when samples contain rare units [69], recent studies took benefit of it to test phytochemical dissimilarity between plant tissues [70] or temporal variations [10]. For each crop cover, we bootstrapped 1000 plant communities and tested linear models between pest occurrence and phytochemical abundance or dissimilarity. Furthermore, the occurrence of pests between crop covers was compared with estimated marginal means (function ‘emmeans’, one-sided pairwise comparison with ‘Benjamini and Hochgeb’ *p*-value adjustment) of generalized linear models (function ‘glm’, Poisson distribution).

## 3. Results

### 3.1. Phytochemistry varies between cultivars and species

**Figure 4.**
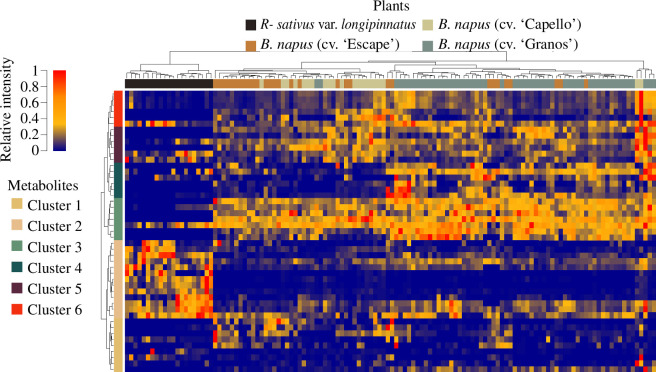
Variations of plant metabolome across cultivars and species. Hierarchical clustering indicates that sampled plants (columns) aggregate differentially according to the production of 47 features (rows) retrieved from random forest analyses.

Random forest analyses demonstrate that the metabolome is powerful in predicting to which species or cultivars plants belong (0 ± 0%, 1 ± 1%, 8 ± 2% and 16 ± 3% error rates in classification for radish, ‘Granos’, ‘Escape’ and ‘Capello’, respectively). In figure 4, plant species and cultivars discriminate according to the 47 features with the highest importance measures in 990 random forest analyses (*n* = 1000). Radish is characterized by the downregulation of gluconapin (metabolomic cluster 3) and, meanwhile, the upregulation of metabolomic cluster 2 including terpenoids, diholosides, and glycosylated hydroxycinnamic acids. Among *B. napus*, the cultivar ‘Granos’ contains a large amount of 4-caffeoylquinic acid and, to a lesser extent, glucoalyssin (metabolomic cluster 4) compared with ‘Capello’ and ‘Escape’. The ‘Capello’ cultivar differs from ‘Escape’ based on a higher production of progoitrin and glucobarbarin (metabolomic cluster 5). Because species and cultivars can be considered as chemotypes, our results encourage one to dissect the relationships between pest infestation and phytochemistry according to plant-specific approaches.

### 3.2. Natural compounds driving pest infestation

Overall, we recorded 141 positive relationships between relative intensities of features and herbivory rates, while only 37 were negative (electronic supplementary material, figure S1). This ratio is relatively well conserved for the 24 relationships shown in figure 3 based on the highest correlation coefficients and glucosinolate-targeted approach. Compounds that support the highest correlation coefficients with herbivory rates differ between not only plants but also monoculture and mixed crops for a single cultivar. In total, seven glucosinolates are significantly correlated with pest infestations (figure 3). Four glucosinolates are positively correlated with herbivory rates, including two unknown compounds (*m*/*z* = 416.105 and *m*/*z* = 523.043) and two aliphatic glucosinolates: gluconapoleiferin and glucobrassicanapin (*ρ* = 0.44 and *ρ* = 0.41, respectively). In contrast, two aliphatic glucosinolates are negatively correlated with herbivory rate, that is, gluconapin (*ρ* = −0.58) and glucoraphenin (*ρ* = −0.37) along with the arylaliphatic glucobarbarin (*ρ* = −0.36). Furthermore, figure 3 indicates that two fatty acids (*m*/*z* = 825.205 and *m*/*z* = 311.258) and one flavonoid glycoconjugated with hydroxycinnamic acid (*m*/*z* = 905.234) adversely affect herbivory. In contrast, particular fatty amines/amides, terpenoids and hydroxycinnamic acids can favour pest infestation.

### 3.3. Individual phytochemical makeups have a poor explanatory power on pest infestation

**Figure 5.**
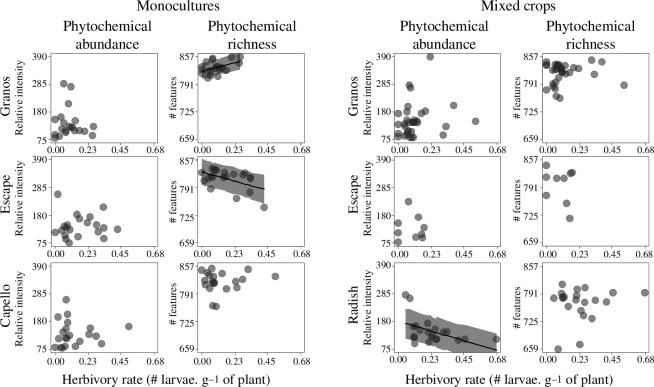
Relationships between pest infestation and phytochemical makeups of individual plants. Phytochemical abundance is the quantitative sum of all features detected in a single plant. Phytochemical richness is the number of different features detected in a single plant. Black lines indicate significant mixed linear models (‘block’ as random factor; *p* < 0.05). Grey areas depict prediction intervals (80%) based on simulated models (*n* = 1000).

Phytochemical abundance was significantly correlated with pest infestation in only one plant, radish (figure 5, *p* = 0.002). In monoculture, while phytochemical richness showed a positive relationship with herbivory rate in ‘Granos’ (figure 5, *p* = 0.021), we observed the opposite pattern in ‘Escape’ (figure 5, *p* = 0.011). In addition to these three case figures, the nine additional relationships between phytochemical makeup and pest infestation were not significant (figure 5, *p* ≥ 0.05).

### 3.4. The phytochemical abundance and dissimilarity of crop covers are two opposite drivers of pest infestation

**Figure 6.**
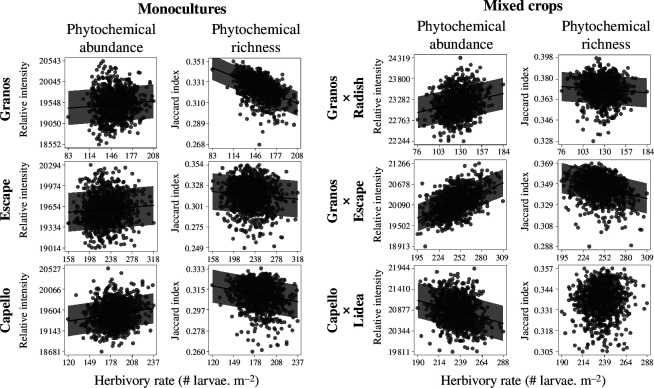
Relationships between pest occurrence and phytochemistry at the plant community level. Each grey dot depicts a bootstrapped plant assemblage (*n* = 1000). Phytochemical abundance is the quantitative sum of all features detected in each plant community. Phytochemical diversity is estimated with β-diversity indicators (average Jaccard dissimilarity index). Black lines and grey areas indicate coefficients of significant linear models (*p* ≤ 0.05) and confidence intervals (80%), respectively.

As shown in figure 6, the phytochemical abundance of crop covers covaried positively with pest infestation, except for mixed crops with ‘Capello’ × ‘Lidea’ where we found a negative relationship (*ρ* = −0.248, *p* ≤ 0.001). Positive correlations were higher in the two other mixed crops ‘Granos’ × ‘Escape’ (*ρ* = 0.488, *p* ≤ 0.001) and ‘Granos’ × ‘Radish’ (*ρ* = 0.206, *p* ≤ 0.001) as compared with monocultures with ‘Granos’ (*ρ* = 0.070, *p* = 0.026), ‘Escape’ (*ρ* = 0.072, *p* = 0.022) and ‘Capello’ (*ρ* = 0.164, *p* ≤ 0.001). For phytochemical dissimilarity, we observed the opposite pattern in five crop covers over six. Negative relationships were ranked as following: ‘Granos’ (*ρ* = −0.467, *p* ≤ 0.001), ‘Granos’ × ‘Escape’ (*ρ* = −0.285, *p* ≤ 0.001), ‘Capello’ (*ρ* = −0.183, *p* ≤ 0.001), ‘Escape’ (*ρ* = −0.086, *p* = 0.007) and ‘Granos’ × ‘Radish’ (*ρ* = −0.077, *p* = 0.014). Only one crop cover, ‘Capello’ × ‘Lidea’, did not show significant covariations between phytochemical dissimilarity and pest infestation of crop covers (figure 6).

## 4. Discussion

In this study, we bridge chemical and community ecology with recent developments in metabolomics to harness the agroecological potential of phytochemistry. Based on a manipulative field experiment, we stress how phytochemical diversity varies between crop covers and, more importantly, affects a specialist pest of brassicaceous crops, *P. chrysocephala*. According to classic hypotheses in plant defence theory, we discuss different relationships between herbivory and phytochemistry, whether it is considered at the compound, the plant or the crop cover scale. Thereby, this study provides a big picture of covariations between herbivory and phytochemical diversity, even if we do not address causal relationships. This research line paves new directions to expand morphological-based functional frameworks used in agroecological research.

### 4.1. Natural compounds driving pest infestation

In our field study, the plant metabolome was powerful in predicting cultivars regardless of the environmental stochasticity experienced by the plants. In other words, cultivars can be viewed as chemotypes based on the stability of phytochemical signatures. Discrimination of chemotypes may help to fill current gaps in knowledge in functional screening of oilseed rape resistance against herbivores [71,72]. In line with initial expectations, we confirm that the cultivars ‘Granos’ and ‘Capello’ display a relatively low level of infestation compared with the cultivar ‘Escape’ and *R. sativus* var. *longipinnatus*, i.e. two plants generally considered as trap plants (electronic supplementary material, figure S2). Taken together, our results indicate that oilseed rape resistance against *P. chrysocephala* depends on intraspecific genetic diversity and that such resistances likely involve phytochemistry.

Among the 178 significant relationships between metabolites and herbivory rate, 80% were positive. This ratio reflects the high degree of specialization of *P. chrysocepahala* in brassicaceous crops and thus corroborates the hypothesis of ‘phytochemically mediated coevolution’ in our pathosystem. A large body of evidence demonstrates that specialist pests, like the cabbage stem flea beetle, have evolved counterdefences to use glucosinolates as feeding stimulants [23] or for their own defences [24,25]. This study shows more particularly that *P. chrysocephala* takes advantage of four of them in modern cultivars of oilseed rape. While brassicanapin, gluconapoleiferin and one unannotated glucosinolate (*m*/*z* = 523.043) were chemotype specific, another glucosinolate (*m*/*z* = 416.105) supported positive relationships with pest not only in ‘Granos’ and ‘Escape’ but also in radish despite important concentration differences between these three genotypes. On the contrary, glucobarbarin, glucoraphenin and gluconapin were negatively correlated with herbivory rates. Our results are consistent with molecular investigations demonstrating that *P. chrysocephalla* has evolved detoxification enzymes with narrow specificities against particular glucosinolates [73]. Beyond glucosinolates and antiherbivore compounds, this study indicates that compounds that favour pests could provide additional dimensions to screen agroecological cultivars preventing pest infestation. For example, this study encourages the adoption of cultivars downregulating hydroxycinnamic acids, such as methyl caffeate and 5-cafeoylquinic acid, or fatty acids such as (6Z,9Z,12Z,15Z)-octadecatetraenoic acid and (10E,12Z,15Z)-9-hydroxyoctadecatrienoic acid. Although the use of untargeted metabolomics to screen resistant cultivars is still at its early age [74], this approach is promising for taking advantage of a wide array of phytochemical traits that jointly regulate herbivory. This may be of first importance to forestall the emergence of pest resistance against a few molecules and, meanwhile, to integrate pest communities attacking plants concomitantly.

### 4.2. Phytochemical makeup of individual plants has poor explanatory power on pest infestation

Contrary to our initial expectation, the hypothesis of ‘synergistic effect’ failed to explain the pest infestation of individual plants. Based on a meta-analysis, Richards *et al*. [29] pointed out that specialist herbivores, like *P. chrysocephala*, are less affected by synergistic effects compared with generalists. To some extent, an increase in phytochemical mixture can even favour the nutrient balance of insects feeding on narrow ranges of host plants [75]. However, from a critical viewpoint, it remains challenging to conclude about synergistic effects when such effects can emerge within or between various ontological groups of chemicals like superclasses, classes or subclasses [28,29]. In our case, even the particular diversity within the chemical class of glucosinolate did not show any significant patterns with pest infestation (electronic supplementary material, figure S3). Moreover, we cannot exclude that synergistic effects could be overruled by antagonist relationships, i.e. when multiple compounds reduce their efficacy of each other [28]. In addition, the poor explanatory power of synergistic effects could also indicate that bipartite interactions between focal plants and single pests are not the most relevant to interpret entire phytochemical makeups. In a recent study, Whitehead *et al*. [76] suggested that phytochemical diversity of individual plants is mainly driven by multiple pest infestations occurring on the community scale.

### 4.3. Phytochemical abundance and dissimilarity on the crop cover scale support opposite patterns in pest infestation

The idea that pest populations are partially regulated by plant resources, also known as bottom-up pest control, originates from a seminal experiment conducted on flea beetles attacking brassicaeous crops [77]. This assertion grounded in community ecology came just after the same author coined the ‘associational resistance*’* hypothesis based on behavioural assays demonstrating that individual pests perform less when host plants are associated with non-host plants [34]. Five decades after, recent progress in high-throughput metabolomics and large-scale data acquisition offers interesting tools for chemical ecologists aiming to bring together individually centred mechanisms with ecological outcomes and dynamics at the community level [31,78]. With this in mind, our results help to understand how bottom-up pest control arises, at least partially, from the phytochemical mechanisms underlying the hypothesis of ‘associational resistance’.

In our case study, this hypothesis was corroborated by negative relationships between the distribution of *P. chrysocephala* and the phytochemical diversity of the crop covers. Therefore, phytochemistry could provide an interesting perspective to broaden trait-based frameworks generally used to reason biodiversity-based management of crop covers. Although a large body of research interested in associational resistance has focused on interspecific diversity, our results align with recent studies reporting the role of intraspecific diversity in collards [79] and tobacco [80]. This is important to take full benefit of phytochemical diversity in mixed crops [81]. However, to this end, we advocate to account for the pathosystems and agronomic perspectives. First, in opposition to phytochemical diversity, our results show that phytochemical abundance favoured herbivory in five crop covers over six. In the case of specialist pests, such as *P. chrysocephala*, we can reasonably think that appropriate landings in plant communities with high amounts of feeding stimulants can enhance pest settlements and, ultimately, infestation on local patch of plants; see ‘trapping effects’ [77] and ‘appropriate/inappropriate landing’ [82]. In addition, we cannot exclude that generalist pests could perform better in plant communities with high chemodiversity according to diet-mixing patterns [75] or associational susceptibility [83]. Additionally, from agronomic perspectives, trap plants could serve as sinks that can result in associational susceptibility through spill-over effects [84]. Taken together, these effects probably explain why trap plants randomly combined with target crops failed to reduce pest infestation as compared with monocultures with trap crop alone (electronic supplementary material, figure S4). This stresses the need to consider the densities and spatial arrangements of trap plants in agronomic systems that attempt to modulate the phytochemical diversity of crop covers [80,84].

## 5. Conclusion and perspectives

To sum up, we hope that this study will help to integrate phytochemical traits in functional diversity of cropping systems. We argue that much more remains to be done to better understand joint effects of phytochemistry and classic traits of functional ecology, like plant height or biomass, on crop cover infestation. Our results highlight how basic research interested in the integration of multi-scale relationships between pests and phytochemical diversity opens two complementary avenues to extend functional frameworks used in agroecology. First, a surge in interest is needed for large-scale screening of antiherbivore resistance of cultivars. The development of a database integrating current cultivars and their repositories of plant defensive metabolites may help to select appropriate plants in cropping systems less reliant on insecticides. Furthermore, while this study relies on a punctual survey when the number of pest larvae is supposed to be maximal, we advocate for dynamic surveys to unravel the role of different phytochemical cues regulating pest development. This may help to understand which components of phytochemical diversity affect adult attraction, colonization, oviposition or larval mortality.

Second, from community ecology perspectives, this study points out that crop mixtures are interesting to promote phytochemical diversity and, ultimately, pest control. However, these agroecological strategies raise important questions related to the spatial arrangement of phytochemical diversity and the host range of pests. Parallel to our case study on specialist pest and mixed intercropping, further investigations are needed on generalist pests and spatially organized intercropping (e.g. strips inside crops, trap plants surrounding crops). We cannot exclude that polyphagous pests will perform better in crops providing a large range of diets. More generally, we argue that functional frameworks used to manage phytochemical diversity of crop covers should be designed according to pest behaviour and, more particularly, locomotion and foraging strategies.

## Data Availability

All data of this article are available from the FAIR-aligned repository data.gouv.fr [85]. Supplementary material is available online [86].
